# Review of Pesticide Urinary Biomarker Measurements from Selected US EPA Children’s Observational Exposure Studies

**DOI:** 10.3390/ijerph8051727

**Published:** 2011-05-24

**Authors:** Peter P. Egeghy, Elaine A. Cohen Hubal, Nicolle S. Tulve, Lisa J. Melnyk, Marsha K. Morgan, Roy C. Fortmann, Linda S. Sheldon

**Affiliations:** 1 Human Exposure and Atmospheric Sciences Division, National Exposure Research Laboratory, Office of Research and Development, U.S. Environmental Protection Agency, Research Triangle Park, NC 27711, USA; E-Mails: tulve.nicolle@epa.gov (N.S.T.); morgan.marsha@epa.gov (M.K.M.); fortmann.roy@epa.gov (R.C.F.); sheldon.linda@epa.gov (L.S.S.); 2 National Center for Computational Toxicology, Office of Research and Development, U.S. Environmental Protection Agency, Research Triangle Park, NC 27711, USA; E-Mail: hubal.elaine@epa.gov; 3 Microbiological and Chemical Exposure Assessment Research Division, National Exposure Research Laboratory, Office of Research and Development, U.S. Environmental Protection Agency, Cincinnati, OH 45268, USA; E-Mail: melnyk.lisa@epa.gov

**Keywords:** pesticides, urinary biomarkers, human exposure, residue intake, exposure trends, exposure variability

## Abstract

Children are exposed to a wide variety of pesticides originating from both outdoor and indoor sources. Several studies were conducted or funded by the EPA over the past decade to investigate children’s exposure to organophosphate and pyrethroid pesticides and the factors that impact their exposures. Urinary metabolite concentration measurements from these studies are consolidated here to identify trends, spatial and temporal patterns, and areas where further research is required. Namely, concentrations of the metabolites of chlorpyrifos (3,5,6-trichloro-2-pyridinol or TCPy), diazinon (2-isopropyl-6-methyl-4-pyrimidinol or IMP), and permethrin (3-phenoxybenzoic acid or 3-PBA) are presented. Information on the kinetic parameters describing absorption and elimination in humans is also presented to aid in interpretation. Metabolite concentrations varied more dramatically across studies for 3-PBA and IMP than for TCPy, with TCPy concentrations about an order of magnitude higher than the 3-PBA concentrations. Temporal variability was high for all metabolites with urinary 3-PBA concentrations slightly more consistent over time than the TCPy concentrations. Urinary biomarker levels provided only limited evidence of applications. The observed relationships between urinary metabolite levels and estimates of pesticide intake may be affected by differences in the contribution of each exposure route to total intake, which may vary with exposure intensity and across individuals.

## 1. Introduction

There is currently a renewed emphasis within the U.S. Environmental Protection Agency (EPA) on protecting vulnerable subpopulations, especially children, and understanding children’s exposure to chemicals [1]. Children are exposed to a wide variety of chemicals in their homes, schools, daycare centers, and other surroundings [2]. These chemicals may originate from outdoor sources such as ambient air contaminants or indoor sources such as building materials, furnishings, and consumer products. Among consumer products are pesticides used to control roaches, rats, termites, ants, and other vermin. Because of widespread residential and agricultural use, EPA has extensively studied children’s exposures to pesticides over the past decade, particularly using biomarkers to assess the factors that impact exposure.

Biomarkers have the potential to play an important role in assessing aggregate exposure and informing cumulative risk assessment [3,4]. Biomarkers can be indicators of the body burden of a chemical, reflecting all routes of exposure, as well as inter-individual differences in absorption and metabolism. In human observational studies involving young children, urine is the primary vehicle for biomonitoring, being advantageous over blood in its noninvasiveness, ease of collection, and available quantities [5]. Compared to external concentrations of chemicals, biomarkers are often believed to be more directly related to potential adverse health effects [6,7]. Biomarkers, however, are not without their shortcomings, with disadvantages related to the cost and precision of the measurements, uncertainties in the fraction of the absorbed compound that is eliminated, and ambiguity in the overall relationship between biomarkers and external exposures [8,9]. Biomarkers in urine, in particular, are relatively short-lived and highly variable in concentration. Under these conditions it is often more difficult to relate biomarker concentrations to either health outcomes or exposures.

The relationship between a biological marker concentration and external exposure is influenced by factors related both to the environment and to human physiology as well as timing of the exposure and collection of the measurement. Factors related to the environment include spatial and temporal variability in exposure concentrations and effects of the presence of other chemicals [10]. Factors related to human physiology include differences in the rates of absorption, distribution, metabolism, and excretion, both over time and across individuals [11]. Biological monitoring performed concurrently with exposure monitoring may be used to investigate the influence of environment- and subject-specific factors on the relationship between the two and to evaluate the relative contribution of the various exposure routes to the observed biomarker levels [12–14].

The objective of this article was to review the kinetic parameters of organophosphate and pyrethroid pesticides and compare measurements of biomarkers of these pesticide exposures among children across several studies. The results and analysis presented in this article were developed as a component of a larger analysis of studies conducted and funded by EPA to identify key factors that influence children’s exposure to pesticides along all relevant pathways. The report *Important Exposure Factors for Children: an Analysis of Laboratory and Observational Field Data Characterizing Cumulative Exposure to Pesticides* [15] compares results across studies to identify trends and reveals areas where progress has been made in reducing uncertainties. This article consolidates urinary biomarkers measurements from both large- and small-scale observational studies to identify trends that otherwise may not be apparent in analyses of the individual studies.

## 2. Background

### 2.1. Review of the Toxicokinetics of Organophosphate and Pyrethroid Pesticides

Organophosphate (OP) pesticides are typically composed of an *O,O*-dialkyl substituted phosphate, phosphorothioate, or phosphorodithioate moiety and an organic group [16]. The organophosphates are more water soluble and have higher vapour pressures than the chlorinated pesticides that they replaced [17]. Pyrethroids are synthetic analogs of the naturally occurring pyrethrins, which are esters of a substituted cyclopropanecarboxylic acid and a substituted cyclopentenolone alcohol. Variations of the substituent groups on the acid and alcohol moieties increase stability and decrease volatility [18]. Some understanding of organophosphate and pyrethroid pesticide toxicokinetics is necessary to meaningfully compare the urinary biomarker concentrations presented here with environmental and dietary concentrations. Despite extensive usage of pesticides, remarkably little information on kinetic parameters describing absorption and elimination in humans is available from the scientific literature. Reported parameters extracted from the literature are summarized in Table 1. While not specifically addressed in this article, pyrethroids generally have lower vapor pressures and higher octanol/water partition coefficients than organophosphates pesticides, resulting in a tendency to favor the particulate phase at room temperature and to partition into organic matter (such as housedust). This not only affects translocation inside the home, but also can influence transfer mechanisms important for exposure as well and absorption into the body.

### 2.2. Absorption

Following inhalation, absorption takes place in the alveolar region of the respiratory tract, where contaminants diffuse passively through the thin alveolar membrane and are absorbed into the bloodstream [30]. Inhalation studies with a variety of gases have shown that even the most efficiently absorbed low molecular weight, highly water soluble compounds rarely exceed 70% uptake [31]. No studies reporting the fraction of organophosphate pesticides absorbed through inhalation were found, but Oberst *et al*. [32] reported that close to 70% of sarin, a structurally related organophosphate cholinesterase inhibitor was absorbed. For pyrethroids, Leng *et al.* [28] reported that only about 16% of cyfluthrin was absorbed through inhalation, but no estimates for other pyrethroids are available.

The importance of the dietary contribution to aggregate exposure among infants and young children is well known [25], but few studies have investigated what fraction of ingested pesticide residue is absorbed. For organophosphates, Nolan *et al.* [19] estimated 70% absorption of chlorpyrifos based on urinary 3,5,6-trichloro-2-pyridinol (TCPy), whereas others estimated 60% to 93% absorption based on dialkylphosphate (DAP) metabolites [20,22]. Diet reportedly affects absorption [33]. As for pyrethroids, Woollen *et al.* [29] estimated that 27–57% of cypermethrin was absorbed, while Eadsforth and colleagues [34,35] estimated 45-49% and 72–78% for the *cis* and *trans* isomers, respectively.

Dermal absorption in the residential environment is typically believed to be low due to loss by washing, evaporation, or exfoliation [36]. For organophosphate pesticides, absorption of chlorpyrifos was estimated, based on its primary metabolite TCPy, to be 1.28% of an applied dose of 4 mg/cm^2^ (over 12–20 h) [19], and 1.2% and 4.3% of applied doses of 0.15 and 0.05 mg/cm^2^ (over 4 h), respectively [21]. Absorption of both chlorpyrifos and diazinon was estimated to be about 1% of applied doses of about 0.4 and 1.3 mg/cm^2^ (over 8 h), respectively, based on DAP metabolites [20,22]. A much higher absorption fraction was reported for malathion applied at a much lower dose (4 μg/cm^2^) [36], suggesting that absorption may be inversely related to applied dose. Large differences were reported by anatomical area [37] and among individuals [36]. For pyrethroids, Bartelt and Hubbell [26] found only about 2% of applied permethrin to be absorbed within 24 h. Wester *et al.* [38] observed that approximately 2% (forearm) and 7.5% (scalp) of radiolabeled pyrethrin, applied at a μg/cm^2^ level, were absorbed. ATSDR [24] has concluded that for pyrethroids in general, <2% of the applied dermal dose is absorbed, at a rate much slower than the rate of absorption by the oral or inhaled routes. However, since the applied dermal dosing studies were performed using relatively high loadings and since absorption decreases with increasing dermal loadings, the low values obtained for dermal absorption may be an artifact of study design and may underestimate absorption at the very low levels measured in field studies [27]. On the other hand, particle-bound residues, particularly pyrethroids, may also have a reduced potential for dermal absorption, as a consequence of being bound to the particle.

Due to the paucity of available information on absorption from human studies, simple default values based on human studies, animal studies, and conservative assumptions are often required. For young children (ages 1–6) the following route-specific absorption is often assumed: 50–100% for inhalation, 50% for ingestion, and 1–3% for dermal. In addition, a daily intake of 100 mg of house dust is assumed for indirect ingestion [39]. These absorption assumptions are a source of substantial uncertainty in route-specific intake estimates.

### 2.3. Distribution and Metabolism

Once in the bloodstream, organophosphate and pyrethroid pesticides are rapidly distributed and metabolized. With OP pesticides, hydrolytic cleavage of the ester bond yields one dialkyl phosphate (DAP) metabolite and one organic group moiety [16]. Dimethyl OPs (including malathion, phosmet, and azinphos-methyl) produce dimethyl metabolites and diethyl OPs (including chlorpyrifos and diazinon) produce diethyl metabolites [40]. The organic group metabolites 2-isopropyl-6-methyl-4-pyrimidinol (IMP) and 3,5,6-trichloro-2-pyridinol (TCPy) are the primary metabolites for diazinon and chlorpyrifos, repectively.

Once absorbed, pyrethroids are rapidly metabolized by hydrolysis of the central ester linkage and oxidation of both acid and alcohol moieties, converting both moieties to carboxylic acids. Most of the current use pyrethroids have an alpha-cyano-3-phenoxybenzoxy or a 3-phenoxybenzoxy group as the alcohol moiety and produce 3-phenoxybenzoic acid (3-PBA) as the ester cleavage metabolite [41]. The 3-phenoxybenzoic acid (3-PBA) metabolite is common to 10 of the 18 pyrethroids registered in the United States, including permethrin, cypermethrin, deltamethrin, esfenvalerate [42]. Other benzoic acid metabolites analogous to 3-PBA are more specific and include 4-fluoro-3-phenoxybenzoic acid (4F3PBA) from cyfluthrin and 2-methyl-3-phenylbenzoic acid (MPA) from bifenthrin. These are not necessarily terminal metabolites; for example, as much as 38% of 3-PBA has been reported by Woollen *et al.* [29] to undergo further oxidation to 3-(4′-hydroxyphenoxy) benzoic acid (4OH3PBA). Cleavage of current pyrethroids also typically produces dihalovinyl-substituted chrysanthemic acid derivatives. The chrysanthemic acid derivative *cis*-2,2-dibromovinyl-2,2-dimethyl-cyclopropane-1-carboxylic acid (DBCA) is specific to deltamethrin while the *cis*- and *trans*-isomers of 2,2-dichlorovinyl-2,2-dimethyl-cyclopropane-1-carboxylic acid (DCCA) are common to permethrin, cypermethrin, and cyfluthrin.

### 2.4. Excretion

Both the OP and pyrethroid metabolites are rapidly eliminated in urine. Elimination appears to follow first-order kinetics, with elimination half-times in humans ranging from 2 to 41 h for OPs and from 6.4 to 16.5 h for pyrethroids, depending on both the compound and the route of exposure [21,22,24]. The elimination half-life of about 8 h reported for 3-PBA among workers exposed to cypermethrin [43] suggests that 88% of the metabolite is excreted within the first 24 h following exposure.

Time to peak excretion of urinary OP pesticide metabolites depends on the route of absorption [20–22]. Peak excretion is observed to occur 6 to 24 h later when exposure is by the dermal route compared to when exposure is by the oral route, largely because of route-specific differences in absorption following exposure. Peak excretion may occur as late as 48 h following dermal exposure, as observed among volunteers performing scripted activities featuring extensive contact with a treated surface [44]. Extended peak excretion times suggest that chlorpyrifos may be retained by the skin and may remain systemically available for prolonged periods [21].

## 3. Methods

### 3.1. Observational Exposure Measurement Studies

Several studies were conducted or funded by EPA over the past decade to investigate children’s exposure to environmental contaminants (Table 2). The studies, which included measurements of common residential use pesticides in environmental exposure media as well as in biological media, included: Minnesota Children’s Pesticide Exposure Study (“MNCPES”); Children’s Total Exposure to Persistent Pesticides and Other Persistent Organic Pollutants (“CTEPP”); Biological and Environmental Monitoring for Organophosphate and Pyrethroid Pesticide Exposures in Children Living in Jacksonville, Florida (“JAX”); Center for the Health Assessment of Mothers and Children of Salinas Quantitative Exposure Assessment Study (“CHAM-QEA”); Children’s Pesticide Post-Application Exposure Study (“CPPAES”); Pilot Study Examining Translocation Pathways Following a Granular Application of Diazinon to Residential Lawns (“PET”); Dietary Intake of Young Children (“DIYC”). While not focused on children, the National Human Exposure Assessment Survey in Arizona (“NHEXAS-AZ”) also provided useful data.

The studies took place in private residences and in child care centers and have been grouped as either large observational field studies (NHEXAS-AZ, MNCPES, CTEPP) or small pilot-scale observational studies (JAX, CPPAES, DIYC, and CHAM-QEA). The large observational field studies had a regional focus. A broad suite of chemical contaminants, including organophosphate and pyrethroid pesticides and their metabolites, were typically measured in multiple environmental media and in urine. Some of the small pilot-scale studies included measurements of multiple chemicals in multiple media in locations either with year-round residential pesticide use (JAX) or in close proximity to agricultural fields (CHAM-QEA). Other pilot-scale studies focused on a single compound (CPPAES, DIYC, PET).

Information on recruitment was reported in detail in articles presenting results from the individual studies. All studies involving children were observational research studies, as defined by the regulatory requirements set forth in EPA’s human subjects regulations (40 CFR Part 26.402). All study protocols regarding recruitment and treatment of participants, including procedures to obtain the assent of the children and informed consent of their parents or guardians, were reviewed and approved by independent institutional review boards (IRB) and reviewed by the EPA to assure compliance with the Federal Policy for the Protection of Human Subjects (“the Common Rule”).

### 3.2. Urine Sample Collection

All urine samples were collected exclusively at the children’s homes except for the CTEPP study, in which urine samples were also collected at their daycare centers. Urine collection preceded and followed outdoor turf applications in the PET study and routine professional indoor applications in the DIYC and CPPAES studies (Table 2). Spot urine samples, mainly first morning voids, were collected using age-appropriate methods including under-toilet seat bonnet (CTEPP, PET), collection cup (NHEXAS-AZ, MNCPES), diaper insert (DIYC), and “potty chair” (CPPAES).

Sample collection was performed by the children’s caregivers following protocols provided by the investigators. Chemical analysis of urinary pesticide metabolites in nearly all included studies was performed by the National Center for Environmental Health of the Centers for Disease Control and Prevention (CDC) in Atlanta, GA, using validated tandem mass spectrometry techniques [42,56–58]. Chemical analysis for the CTEPP study was performed by Battelle Memorial Institute using validated gas chromatography/mass spectroscopy techniques [59]. Chemical analysis for the DIYC was performed by RTI International using CDC methods.

### 3.3. Pesticides and Their Metabolites

Only selected organophosphate and pyrethroid metabolites that were measured in several of the studies of interest, namely, TCPy, IMP, and 3-PBA, are presented in this article. Several other insecticides and their metabolites were also measured in the studies (Table 2) but are not included in this analysis.

### 3.4. Statistical Analysis

All statistical analyses were performed using SAS version 8.0. Summary statistics generated for urinary biomarkers common to several studies were reviewed and compared. Spot measurements of urinary pesticide biomarkers among children 6 to 12 years old from the 1999–2000 and 2001–2002 cycles of the National Health and Nutrition Examination Survey (NHANES) [60], an ongoing assessment of the exposure of the U.S. population to environmental chemicals, were also included for comparison.

#### 3.4.1. Estimates of Variance Components

The relationship between a single urinary metabolite measurement and the mean of a series of measurements from the same participant was evaluated among a subset of non-composited CTEPP samples collected over 48 hours. The reliability of a single measurement in representing a true longer-term average is a function of the variability of a series of measurements [61]. More specifically, it is a function of the magnitude of the intra-person component of variance with respect to the total variance, or the intraclass correlation coefficient of reliability (ICC). An ICC of at least 0.80 is generally considered to indicate good reliability [61], signifying that the values are consistent from sample-to-sample and that a single measurement sufficiently represents the average of the series of measurements over a specific time period.

The following equation was used to estimate the intraclass correlation coefficient of reliability:

$$\text{ICC}={\hat{\sigma }}_{B}^{2}/({\hat{\sigma }}_{B}^{2}+{\hat{\sigma }}_{w}^{2})$$

where the inter-person variance σ*_B_*^2^ and the intra-person variance σ*_W_* ^2^ were estimated from a random effects model using mixed effects regression. Between- and within-person fold-ranges (_B_R_0.95_ and _W_R_0.95_) were estimated following Rappaport [62] as:

$${}_{B}R_{0.95}=e^{3.92{\hat{\sigma }}_{B}}\;\text{and}\;\;{}_{W}R_{0.95}=e^{3.92{\hat{\sigma }}_{W}}$$

Variance estimates were repeated with urine values adjusted for specific gravity of the sample (SG_sample_) using a target value (SG_target_) of 1.022 [63] and the following equation [64]:

$$\text{SG}-\text{adjusted}\;\text{value}=\text{value}\times ({\text{SG}}_{\text{target}}-1.000)/({\text{SG}}_{\text{sample}}-1.000)$$

#### 3.4.2. Estimates of Route-Specific Exposure

Route-specific exposure and subsequent intake was estimated using CTEPP data by combining concentrations measured in exposure media with rudimentary individual-level time activity information and default exposure factors [47,65].

## 4. Results and Discussion

### 4.1. Summary Statistics for Urinary Biomarker Levels

**Figure 1.**
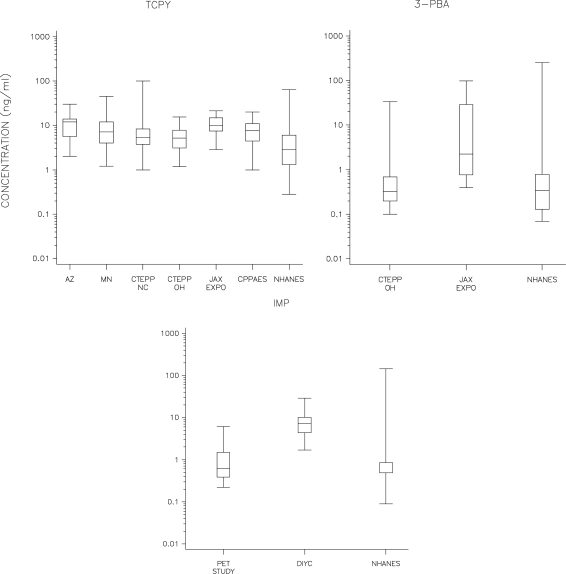
Box-and-whisker plots comparing the urinary TCPy, 3-PBA, and IMP concentrations across studies. NHANES results (6–12 year olds) included for comparison.

Several pesticide metabolites were frequently detected. The chlorpyrifos metabolite, TCPy, was detected above the LOD in over 90% of the children’s urine samples in all listed studies. The pyrethroid metabolite, 3-PBA, was detected at frequencies of over 60% in the CTEPP-OH samples and 100% of the JAX samples, the only two studies in which it was measured. IMP was detected at 77%, 100%, and 0% in the PET, DIYC, and JAX studies, respectively. All measurements of IMP in CTEPP-OH were deemed questionable due to analytical interferences and excluded from this analysis. The detection frequency and the concentrations at the median and 95th percentiles for each urinary metabolite are presented by study in Table 3. Distributions of the urinary metabolite concentrations are depicted with box-and-whisker plots in Figure 1. The median urinary TCPy concentrations were fairly similar across studies, with a fold range of only about 2.4 between the lowest (5.1 ng/mL for CTEPP-NC) and the highest (12.0 ng/mL for NHEXAS-AZ) studies. The median urinary TCPy concentrations in CPPAES, a study with known and monitored applications of chlorpyrifos, did not differ substantially from the medians in the large-scale studies. Among the large-scale studies, there was little difference in urinary TCPy concentrations measured in the North Carolina and Ohio segments of CTEPP, but the concentrations from Minnesota and Arizona were distinctly higher (all unweighted). Higher levels in the Minnesota and Arizona studies may reflect the greater use of chlorpyrifos at the time that the two studies were conducted, and particularly with MNCPES, an intentional oversampling of pesticide-using households [46]. The median TCPy values observed in all EPA studies (ranging from 5.1 to 12 ng/mL) were higher than median for children under 12 years old in the combined 1999–2002 NHANES release (2.8 ng/mL). The median TCPy values were also higher than those reported for children 1–6 years of age (n = 60) in eastern North Carolina farm-worker households (2.47 ng/mL, [66], on par with the median of 9.1 ng/mL reported for 13 children aged 2–5 years from Washington State [67], and below the geometric mean of 16 ng/mL reported for 116 children under the age of 16 years living in Iowa farm and non-farm households [68].

The median urinary concentrations varied more dramatically for 3-PBA and IMP than for TCPy. The median 3-PBA level measured in JAX (2.2 ng/mL), in a location with elevated rates of pesticide use [69], was more than seven times higher than the median in CTEPP-OH (0.3 ng/mL). The median level in CTEPP-OH is consistent with levels among elementary school-age children in and around Seattle, WA (0.45 ng/mL) [70], among children in the population-based NHANES survey in the U.S. (0.32 ng/mL) [60] and GerES IV in Germany (0.29 ng/mL) [71]. For IMP, the median level in the DIYC study (7.1 ng/mL) was ten times higher than in the PET study (0.62), reflecting that measurements followed indoor pesticide applications in the former and outdoor applications in the latter. Measurements from both studies fell within the range found in NHANES.

The box-and-whisker plots presented in Figure 1 are also useful for comparing the different metabolites against each other. Across studies, urinary TCPy concentrations (with medians ranging from 5.1 to 12) appear to be about an order of magnitude higher than the urinary 3-PBA concentrations (medians ranging from 0.3 to 2.2). Concentrations of IMP (medians ranging from 0.62 to 7.1) were roughly in between the concentrations of 3-PBA and TCPy, with concentrations in DIYC in the TCPy range. The relative magnitude of TCPy *versus* 3-PBA concentrations is similar to that found in the nationwide NHANES (2.8 *versus* 0.34) and among children in North Carolina farm-worker households (2.47 *versus* 0.07) [66]. This difference suggests that the study populations experienced greater overall exposure to chlorpyrifos (and environmental TCPy) than to the pyrethroids that are metabolized to 3-PBA (and environmental 3-PBA). However, since chlorpyrifos and diazinon were deregistered by the EPA for most residential uses in 2000 and 2004, respectively [72,73], there is an expectation that non-dietary organophosphate exposures have decreased over time, a trend recently documented among heavy residential pesticide users [74,75].

### 4.2. Evidence of Pesticide Applications in Urinary Concentrations

**Figure 2.**
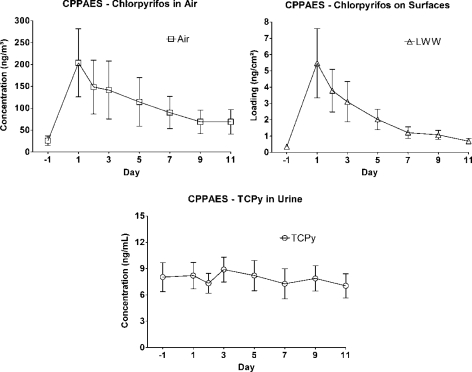
Time profiles for chlorpyrifos in environmental media and TCPy concentrations in urine for all children in the CPPAES following crack and crevice treatment.

Measurements in several studies allowed investigation of the utility of urinary biomarker levels in providing evidence of recent pesticide applications. Biomarker confirmation of pesticide application was stronger with 3-PBA and IMP than with TCPy. The median level of 3-PBA in CTEPP (0.32 ng/mL) was similar to that in NHANES (0.34 ng/mL), but the median 3-PBA value among children in JAX (2.2 ng/mL) was about seven times higher (Table 3). The distribution of levels of IMP from the DIYC study (median = 7.1 ng/mL) was about an order of magnitude higher than the distributions from the PET (median = 0.62 ng/mL) or NHANES (median < 0.7 ng/mL) studies. The relative magnitudes of the urinary IMP levels is in concordance with expectations based on the type of applications, as the participants of the DIYC study performed thorough indoor applications of diazinon while the participants in the PET study applied the insecticide outdoors on turf. Evidence of application, however, was absent in a study following crack and crevice applications of chlorpyrifos in the CPPAES study [53]. In that study, the intensity of application was described as either high (n = 7) or low (n = 3), based on the total amount of active ingredient dispensed. Although mean air concentrations resulting from “high” applications were five orders of magnitude higher than those resulting from “low” applications, the urinary TCPy concentrations were not much different for the children in the high *versus* low application groups [53]. Crack and crevice type applications of chlorpyrifos at these homes did not substantially increase the children’s urinary TCPy concentrations. In fact, the median urinary TCPy concentration for children in the “high” application group was higher one day before application than on the first two days following application. Furthermore, the concentration-time profiles for urinary TCPy levels did not mirror the environmental concentration time profiles (Figure 2). The lack of an observable effect on urine concentrations may be related to exposure to environmental TCPy or to chlorpyrifos and TCPy through the dietary pathway [70,76], but neither of these potential contributions was evaluated in the study.

**Figure 3.**
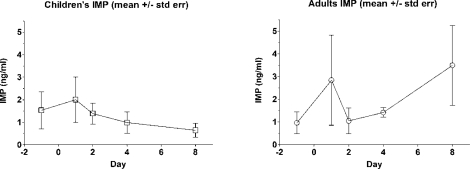
Time-concentration profile for urinary IMP measurements among child and adult PET study participants following an outdoor granular turf pesticide application.

Change in urinary metabolite levels was also investigated with diazinon in the PET study with lawn applications [54] and the DIYC study with indoor applications. While no statistically significant difference in pre- and post-application urinary IMP concentrations was reported by the authors of the PET study, the time-concentration profile appears to show an observable decay in children’s urinary biomarker concentrations in the eight days following the outdoor lawn application (Figure 3). The pattern among adults is not consistent with that among children, perhaps reflecting differences in exposure patterns between adults and children [77]. IMP concentrations in the first morning voids (FMV) of the DIYC children did not decay steadily over time, but behaved similarly to the adults in the PET study (data not shown). The difference between indoor and outdoor application may be a factor in the pattern changes.

### 4.3. Temporal Variability in Biomarker Measurements

Spot urine measurements over 48 hours among CTEPP participants reporting a recent application of any pesticide (within seven days of field monitoring) show large sample-to-sample variability and large differences among individuals (Supplemental Figure S-1). Adjustment of urinary metabolite values by specific gravity did not meaningfully reduce within-person variability of TCPy. Intraclass correlation coefficients of reliability (ICC) and the within- and between-person geometric standard deviations (GSD) for urinary TCPy and 3-PBA concentrations in NC and OH children in the CTEPP study are provided in Table 4.

Reliability is a function of both interpersonal and intrapersonal variability and describes the degree to which a randomly selected single measurement represents the average of a set of measurements on a given individual over a specific time period [78]. The ICC can range from zero to one, with values near one indicating high reliability. A value of 0.80 is typically considered the benchmark above which a single measurement for an individual adequately represents his or her average. As an additional measure of variability, the between- and within-person R_0.95_, representing the fold-range containing the middle 95% of the participant-specific mean concentrations (_b_R_0.95_) or of the repeated measurements for a given participant (_w_R_0.95_), respectively, were estimated. The urinary 3-PBA concentrations were slightly more consistent over time than the TCPy concentrations, as evidenced by a higher ICC. For the Ohio children, the _w_R_0.95_ estimates indicate that 95% of the TCPy measurements for a single individual would be expected to have a 14-fold range, whereas a 5.6-fold range would be expected for the 3PBA measurements. Estimates of ICC suggest that spot sample measurements of neither 3-PBA nor TCPy concentrations exhibit sufficient consistency throughout the 48-h monitoring period to adequately rank the participants’ exposure levels with a single measurement. Furthermore, large intrapersonal variability with respect to interpersonal variability can obscure relationships between a urinary biomarker result and a determinant of exposure (e.g., prior application, route of exposure) through attenuation of the regression coefficient [79,80]. An example of this with pesticide exposures among children can be found in Curwin *et al.* [81], where children’s urinary pesticide levels generally had much higher intra-individual variability and greater attenuation of exposure relationships compared to adults. Similar ICCs (0.59 and 0.49 for NC and OH, respectively) were observed for the herbicide 2,4-dichlorophenoxyacetic acid (2,4-D) in the same cohort [48]; Meeker *et al*. [82] reported similar reliability for the carbaryl metabolite 1-naphthol (ICCs between 0.55 and 0.61) and much lower reliability for TCPy (ICCs between 0.15 and 0.21) among adult men in New England; and Egeghy *et al*. [83] reported somewhat lower reliability for TCPy (0.40) among adults in Maryland. These results point to the need to understand urinary output in order to reduce the observed variability in spot samples [84].

Comparing FMV to other spot samples collected among the same subsample of CTEPP children (data not presented), the geometric mean of the FMV concentrations is substantially higher (45%, p < 0.001) than that of the non-FMV samples for TCPy. Higher FMV concentrations are likely the result of a combination of reduced urinary output at night and the timing of dietary exposures. The same analysis for 3-PBA found FMV to be only negligibly higher (17%, p = 0.40). Since both compounds experienced the same accumulation time in the bladder, the reason for the discrepancy in the results is not entirely clear but may point to differences in exposure routes and patterns for the two parent compounds. A similar analysis was performed in the CHAM-QEA study, where concentrations in the overnight diapers of very young children (6 months to 2 years) were compared to concentrations in spot samples [51]. Median total dialkyl phosphate (DAP) metabolite concentrations were higher in the overnight samples compared to the spot samples (140 *vs.* 100 nmol/L), but the authors reported that the difference was not statistically significant with a Wilcoxon rank sum test. Spot and overnight urine concentrations were significantly correlated in CHAM-QEA, with a Spearman rho of 0.48 (p < 0.01) reported for total DAP metabolites [51].

Kissel *et al*. [67] investigated excretion patterns at various time points throughout the day for two nonspecific and three specific organophosphate metabolites among children approximately 4 years of age. They compared nighttime, FMV, lunchtime, and afternoon samples and reported that the concentrations were highly variable over the course of a single 24-h period. Employing a percent deviation approach to evaluate the correspondence of a single spot sample with average daily results, they found that FMV samples were the best predictors of estimated total daily excretion for the organophosphate metabolites. No similar analysis has been performed for pyrethroid metabolites. For organophosphates these analyses taken together suggest that although FMV samples may slightly overestimate exposure, they still provide a more reliable estimate than what would be available from samples collected at other times of the day.

### 4.4. Relative Importance of Exposure Routes

The relative importance of the dietary ingestion, indirect ingestion, dermal, and inhalation routes of exposure with respect to aggregate intake previously has been investigated through pharmacokinetic (PK) modeling in the DIYC study and through comparisons with biomarker levels in the DIYC, MNCPES, and CTEPP studies. Hu *et al.* [55] identified ingestion as the dominant route of exposure to diazinon among children using PK modeling. In an analysis of the MNCPES chlorpyrifos data, Clayton *et al*. [85] showed, using a mass balance approach, that dietary ingestion was a greater source of intake than inhalation. Similarly, Morgan *et al*. [59] determined that the primary route of intake for chlorpyrifos and permethrin among CTEPP children was dietary ingestion. Dietary ingestion as the dominant route of exposure to pesticides has been supported by other studies. For example, Lu and colleagues [76] substituted organic for conventional food to evaluate the importance of dietary exposure relative to other routes for children in the Seattle, WA area and reported that the contribution of chlorpyrifos from dietary sources far exceeded the contribution from the residential environment.

Curiously, when comparing dietary chlorpyrifos intake to the urinary biomarker concentrations, neither Clayton nor Morgan found strong associations. Clayton [85] reported that the association with urinary metabolite levels was much weaker for the dietary measurements (r = 0.22, p < 0.05) than air concentrations (r = 0.42, p < 0.01). Morgan’s group [47] also reported poor correlation with dietary ingestion (r = 0.12, p > 0.05). These results are similar to the correlation between estimated dietary intake of chlorpyrifos and urinary excretion of TCPy (r = 0.21, p < 0.05) observed among adults in the NHEXAS-Maryland study using a similar algorithm [86]. In a subsequent publication, Morgan’s group reported that the relationship between dietary chorpyrifos and urinary TCPy is obscured by concurrent multi-route exposure to the environmentally occurring metabolite [49]. Differences in absorption and elimination by route of absorption, as discussed in Section 2, may also obscure the relationship. Specifically, a combination of multi-route exposures of varying intensities, differences in time-to-peak-excretion by route, and urine sample collection timing based on oral dosing studies may affect the relationship between external exposure and biomarker.

**Figure 4.**
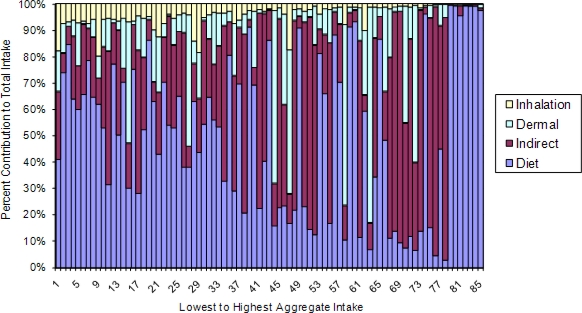
The contributions of inhalation, dermal absorption, indirect ingestion, and dietary ingestion to aggregate intake of *cis*-permethrin in CTEPP-OH (n = 85).

Another factor that may contribute to the poor correlations observed between dietary ingestion and urinary biomarkers may be great variability in indirect intake from hand-to-mouth contact among children [87]. As illustrated in Figure 4 using data from 85 CTEPP-OH participants with a complete set of air, dermal, dust, soil, and food measurements, the contribution of diet to aggregate *cis*-permethrin intake generally decreases (as percent of total contribution) as aggregate intake increases; conversely, indirect ingestion becomes increasingly important with increasing aggregate intake.

More specifically, except at the extremes of the distribution, the contribution from other routes overwhelms that of dietary as aggregate exposure increases. This analysis supports the finding by Lu *et al*. [88] that during episodes of relatively high exposures in the residential environment the contribution of pyrethroids from dietary exposure may be surpassed by other routes. In addition, the DIYC study revealed that the residential use of diazinon can contribute to the dietary route (supplementing the contribution of agricultural sources) through transfer from surfaces to food moderated by the behavioral activities exhibited by the child [55]. The overall result is that while dietary may be the primary source of intake on *average*, the importance of a particular exposure route is not consistent across individuals and may vary with exposure intensity. This may result in an attenuation of the association between the biomarker and a particular route of exposure. Thus, even if intake of environmentally occurring metabolites were negligible, a strong correlation with any one route may be difficult to observe.

## 5. Conclusions

While each pesticide exposure study performed or sponsored by the EPA typically measured the urinary metabolites of several different pesticides, few metabolites were commonly measured in multiple studies. Nonetheless, comparison of those metabolites across studies yields important insights. Distributions of urinary pesticide metabolite levels in children’s observational studies were generally more similar across studies for the major metabolite of chlorpyrifos than for the metabolites of diazinon or the pyrethroid pesticides. Additionally, urinary TCPy concentrations were about an order of magnitude higher than the urinary 3-PBA concentrations. Together these observations suggest that children’s exposures to chlorpyifos (and environmental TCPy) are more uniform across the population than are exposures to the other pesticides that were measured. This may reflect the quantity of chlorpyrifos in use at that period of time, ubiquity of its applications (including as structural termiticides and in agriculture), its transport mechanisms, and its persistence in indoor environments. While measurements of the urinary biomarker TCPy did not reflect known indoor crack and crevice applications of chlorpyrifos, urinary levels of IMP and 3-PBA performed better at capturing outdoor and indoor applications of diazinon and various pyrethroids. With most residential uses of chlorpyrifos and diazinon deregistered shortly after these studies were performed, and with the pyrethroids filling the market void, close attention should be paid to the trend over time in concentrations of urinary pyrethroid metabolites in future studies. Moreover, a greater variety of metabolites should be measured in future studies to better capture the breadth of pesticides that are currently being used.

Although dietary ingestion appeared to be primary route of exposure for both organophosphate and pyrethroid pesticides, the association between estimates of dietary intake and urinary biomarkers is weak. While this is largely the result of the presence of the metabolites in environmental media, there is also evidence that the relationship may be affected by route-specific differences in absorption and by route-specific contributions to total intake that are not consistent across individuals and may vary with exposure intensity. Exposure through multiple routes, as well as the timing of exposure and biomarker collection, may also contribute to the large variability that characterizes urinary biomarker measurements. Successive spot urine measurements showed large sample-to-sample variability in addition to large differences among individuals. Concentrations in first morning void samples were higher than in other spot samples. The difference was much greater for TCPy than for 3-PBA for reasons that are not entirely clear but may point to differences in exposure routes and patterns for the two parent compounds and their environmental metabolites. Perhaps these results can lead to a standard protocol for collection of urine in observational pesticide exposure studies to minimize the variability introduced by these factors.

A currently ongoing study directly addresses the variability of urinary biomarkers of exposure to pyrethroids over time. The study is using statistical and modeling tools and other key information such exposure measurement, activity pattern, and kinetic parameter data to assess the quantitative relationships between urinary biomarkers and human exposures and internal doses to pyrethroids and to their degradates. The project will produce important biomonitoring data on the longitudinal variability and excretion rate of pyrethroid metabolites and other chemicals in urine over time. In addition, this information will be useful in understanding if single or multiple urine samples need to be collected in similar studies, and at frequency of collection would be ideal. The study will provide information regarding the effectiveness of several urine volume adjustment approaches for application in dose estimations using existing biomonitoring data and will determine the feasibility of using exposure reconstruction to estimate exposure in residential settings. Although the study is limited to adult volunteers, it is expected that the information produced will also be useful for the design of future studies of children’s residential pesticide exposure.

## Figures and Tables

**Table S-1.** Limits of detection (ng/mL) for each pesticide metabolite measured in the children’s urine samples by study.

**Study**	**TCPy**	**IMP**	**3-PBA**
NHEXAS-AZ	1.0	NA	NA
MNCPES	1.4	NA	NA
CTEPP-NC	1.0	NA	NA
CTEPP-OH	1.0	NA	0.2
JAX	0.4	2.0	0.5
CPPAES	1.0	NA	NA
PET	NA	0.3	NA
DIYC	NA	1.0	NA

NA, Not Applicable.

**Table 1.** Absorption and elimination characteristics for pesticides and urinary biomarkers of pesticide exposure.

**Compound**	**Absorption of Parent Compound**			**Elimination of Metabolites**		
	**Oral**	**Dermal**	**Inhalation**	**Oral**	**Dermal**	**Inhalation**
Chlorpyrifos	Volunteer studies: 70% of oral dose excreted in urine as TCPy [19], 93% of oral dose excreted in urine as dialkyl-phosphates [20].	Volunteer studies: 1.3% of dermal dose excreted in urine as TCPy [19]. 1% of dermal dose excreted as dialkyl-phosphates [20], 1.2–4.3% of dermal dose excreted as TCPy [21].	No Information.	Volunteer study, 27 h oral [19]. Volunteer study, approx 15.5 h oral [20].	Volunteer study, 27 h dermal [19]. Volunteer study, approx 30 h dermal [20]. Volunteer study, approx 41 h dermal [21].	No Information.
Diazinon	Human oral absorption approx. 60% [22]. Default oral absorption factor of 0.85 [23] .	Human dermal absorption rate: 456 ng/cm^2^/h [22].	No Information.	Human study, 2 h oral [22].	Human study, 9 h dermal [22].	No Information.
Pyrethroids (as a group)	Absorption is incomplete, minimum estimate 40–60%, but first- pass metabolism may underestimate absorption [24].	<2% of the applied dermal dose is absorbed, rate of absorption much slower than by the oral or inhaled routes; may be stored in skin and then slowly released into the systemic circulation [24].	Rapidly absorbed in humans following inhalation, but no estimates of fraction absorbed are available [24].	Elimination appears to follow first-order kinetics, with elimination half-times in humans ranging from 6.4 to 16.5 hours, depending upon the specific pyrethroid and exposure route studied [24].		
Permethrin	Oral absorption factor of 0.70 suggested [25].	Poor dermal absorption: ∼2% of applied dose absorbed/24 h [26]; 7.5% (scalp) and 1.9% (forearm) of applied dose [27].	No Information.	No Information.	No Information.	No Information.
Cyfluthrin	No Information.	No Information.	Human data suggest ∼15% absorption [28].	Human oral dosing produced t-½ of 6.4 h [28].	No Information.	Human ½-lives of 6.9 h (c-DCCA), 6.2 h (t-DCCA), 5.3 h (FPBA) [28].
Cypermethrin	Human volunteer study 27–57% (mean 36%) cypermethrin absorbed [29].	No Information.	No Information.	Human oral dosing, urinary metabolites have mean ½-life of 16.5 h [29].	Human dermal dosing, excretion rates peaked at 12–36 h, mean ½-life was 13 h [29].	No Information.

**Table 2.** Summary of the children’s urinary biomarker collection methods.

**Study**	**N**	**Age Range**	**Sample Collection**	**Collection Strategy**	**Pesticide Application**	**Collection Frequency**	**Analytes of Interest**	**References**
NHEXAS-AZ (subset)	21	5 to 12 years	December 1995 to March 1997	Morning void	No	Once (in 3-day monitoring period)	AM, MDA, 1-Nap, TCPy	[45]
MNCPES	102	3 to 13 years	Summer 1997	Morning void	No	Days 3, 5, and 7 of sampling period	AM, MDA, 1-Nap, TCPy	[46]
CTEPP-NC	130	2 to 5 years	July 2000 to March 2001	Morning void, after lunch, after dinner/before bedtime	No	Over a 48-h period	2,4-D, TCPy, PCP	[47,48]
CTEPP-OH	127	2 to 5 years	April 2001 to November 2001	Morning void, after lunch, after dinner/before bedtime	No	Over a 48-h period	2,4-D, TCPy, 3-PBA, PCP, IMP	[48–50]
CHAM-QEA	20	6 to 24 months	June to October 2002	One overnight and one spot sample	No, but incidental exposure from proximity to farms	Once	DAPs	[51]
JAX	9	4 to 6 years	August to October 2001	Morning void	Yes, indoor as applied by residents	Once	TCPy, IMP, 3-PBA	[52]
CPPAES	10	2 to 4 years	April 1999 to March 2001	Morning void	Yes, indoor professional-applied ‘crack & crevice’	Pre-application and days 1, 2, 3, 5, 7, 9, and 11 post-application	TCPy	[53]
PET	6	5 to 12 years	Spring 2001	Morning void	Yes, outdoor resident-applied turf treatment	Pre-application and days 1, 2, 4, and 8 post-application	IMP	[54]
DIYC	3	1 to 3 years	November 1999 to January 2000	Morning void and other spot samples	Yes, indoor, 2 professional ‘crack & crevice’ and 1 resident treatment	Days 3, 5, and 7 post-application	IMP	[55]

1-Nap, 1-Naphthol; 2,4-D, 2,4-Dichlorophenoxyacetic acid; 3-PBA, 3-Phenoxybenzoic acid; AM, Atrazine Mercapturate; DAPs, Dialkyl phosphate metabolites; IMP, 2-Isopropyl-6-methyl-4-pyrimidinol; MDA, Malathion Dicarboxylic Acid; PCP, Pentachlorophenol; TCPy, 3,5,6-trichloro-2-pyridinol.

**Table 3.** Summary statistics for the pesticide metabolites TCPy, IMP, and 3-PBA measured in the children’s urine samples by study (ng/mL). NHANES results are included for comparison.

	**Study**	**Group**	**n**	**%Det**	**Mean**	**SD**	**GM**	**GSD**	**Min**	**25th**	**50^th^**	**75th**	**95th**	**Max**
TCPy	NHEXAS-AZ	≤12 years	21	100	12	7.6	9.3	2.2	2.0	5.7	12	14	26	30
	MNCPES	All	263	92	9.2	7.7	6.6	2.3	<1.4	4.0	7.2	12	23	45
	CTEPP-NC	All	129	98	7.5	10	5.5	2.1	<1.0	3.8	5.3	8.4	16	100
	CTEPP-OH	All	123	100	5.9	3.5	4.9	1.9	1.2	3.1	5.2	7.8	12	15
	JAX	All	9	100	11	6.4	9.1	2.1	2.9	7.5	9.8	15	21	21
	CPPAES	All	81	93	8.0	4.7	6.4	2.1	<1.0	4.5	7.7	11	18	20
	NHANES	≤12 years	1245	90	4.7	6.1	2.6	3.2	<0.4	1.3	2.8	6.0	15	64
3-PBA	CTEPP-OH	All	126	68	0.81	3.0	0.38	2.6	<0.20	<0.20	0.32	0.69	1.9	34
	JAX	All	9	100	19.6	33	3.9	7.5	0.39	0.76	2.2	29	99	99
	NHANES	≤12 years	679	79	1.4	10	0.36	3.7	<0.10	0.13	0.34	0.78	3.8	254
IMP	PET	All	30	77	1.3	1.6	0.75	2.8	<0.22	0.39	0.62	1.5	5.5	6.2
	DIYC	All	41	100	9.0	6.9	7.1	2.0	1.7	4.4	7.1	10	27	29
	NHANES	≤12 years	1220	15	NC	NC	NC	NC	<0.7	<0.7	<0.7	<0.7	3.0	145

NC, Not calculated.

**Table 4.** Estimated between- and within-person fold-ranges (_b_R_0.95_ and _w_R_0.95_) and Intraclass Correlation Coefficients (ICC) for logged urinary pesticide metabolite concentrations from children in the CTEPP study.

**Metabolite**	**Measure**	**NC Children**	**OH Children**

3-PBA	_b_R_0.95_	–^b^	13
	_w_R_0.95_	–	5.6
	ICC^a^	–	0.69

	_b_R_0.95_	13	11
TCPy	_w_R_0.95_	6.5	14
	ICC	0.65	0.44

a An ICC of 0.80 indicates that a single measurement reliably represents the average of a set of measurements.

b “–” = no data.

## Supplemental Materials

Figure S-1. Concentration *versus* time plots for urinary TCPy measurements among CTEPP-NC and CTEPP-OH participants reporting a recent pesticide application. Urines in panels A and B are without adjustment. Urines in panels C and D are adjusted by specific gravity. Note that not all voids within the 48 hour period were collected.

## Abbreviations and Acronyms

%Det: Percent of samples above detection limit
2,4-D: 2,4-Dichlorophenoxyacetic acid
3-PBA: 3-Phenoxybenzoic acid
4F3PBA: 4-fluoro-3-phenoxybenzoic acid
4OH3PBA: 3-(4’-hydroxyphenoxy) benzoic acid
ATSDR: Agency for Toxic Substances and Disease Registry
CHAM-QEA: Center for the Health Assessment of Mothers and Children of Salinas Quantitative Exposure Assessment Study
CPPAES: Children’s Pesticide Post-Application Exposure Study
CTEPP: Children’s Total Exposure to Persistent Pesticides and Other Persistent Organic Pollutants Study
DAP: Dialkylphosphate
DBCA: 2,2-dibromovinyl-2,2-dimethyl-cyclopropane-1-carboxylic acid
DCCA: 2,2-dichlorovinyl-2,2-dimethyl-cyclopropane-1-carboxylic acid
DIYC: Dietary Intake of Young Children Study
EOHSI: Environmental and Occupational Health Sciences Institute Study
EPA: U.S. Environmental Protection Agency
FMV: First morning void
GM: Geometric mean
GSD: Standard deviation of the geometric mean
ICC: Intraclass Correlation Coefficient
IMP: 2-Isopropyl-6-methyl-4-pyrimidinol
JAX: Biological and Environmental Monitoring for Organophosphate and Pyrethroid Pesticide Exposures in Children Living in Jacksonville, Florida Study
LOD: Limit of detection
Max: Maximum
MDA: Malathion dicarboxylic acid
MDL: Minimum detection limit
Min: Minimum
MNCPES / MN: Minnesota Children’s Pesticide Exposure Study
MPA: 2-methyl-3-phenylbenzoic acid
N: Sample size
NC: North Carolina
NERL: National Exposure Research Laboratory
NHANES: National Health and Nutrition Examination Survey
NHEXAS-AZ: National Human Exposure Assessment Survey in Arizona
OH: Ohio
OP: Organophosphate
25th: 25th percentile
50th: Median/50th percentile
75th: 75th percentile
95th: 95th percentile
PBPK: Physiologically-Based Pharmacokinetic Model
PET: A Pilot Study Examining Translocation Pathways Following a Granular Application of Diazinon to Residential Lawns Study
SD: Standard deviation of the arithmetic mean
TCP: 3,5,6-Trichloro-2-pyridinol
