# Supplementary figures and images for: Review of Pesticide Urinary Biomarker Measurements from Selected US EPA Children’s Observational Exposure Studies
Source: Int J Environ Res Public Health. 2011 May 24;8(5):1727–54. doi: 10.3390/ijerph8051727 (PMC3108137; doi:10.3390/ijerph8051727)

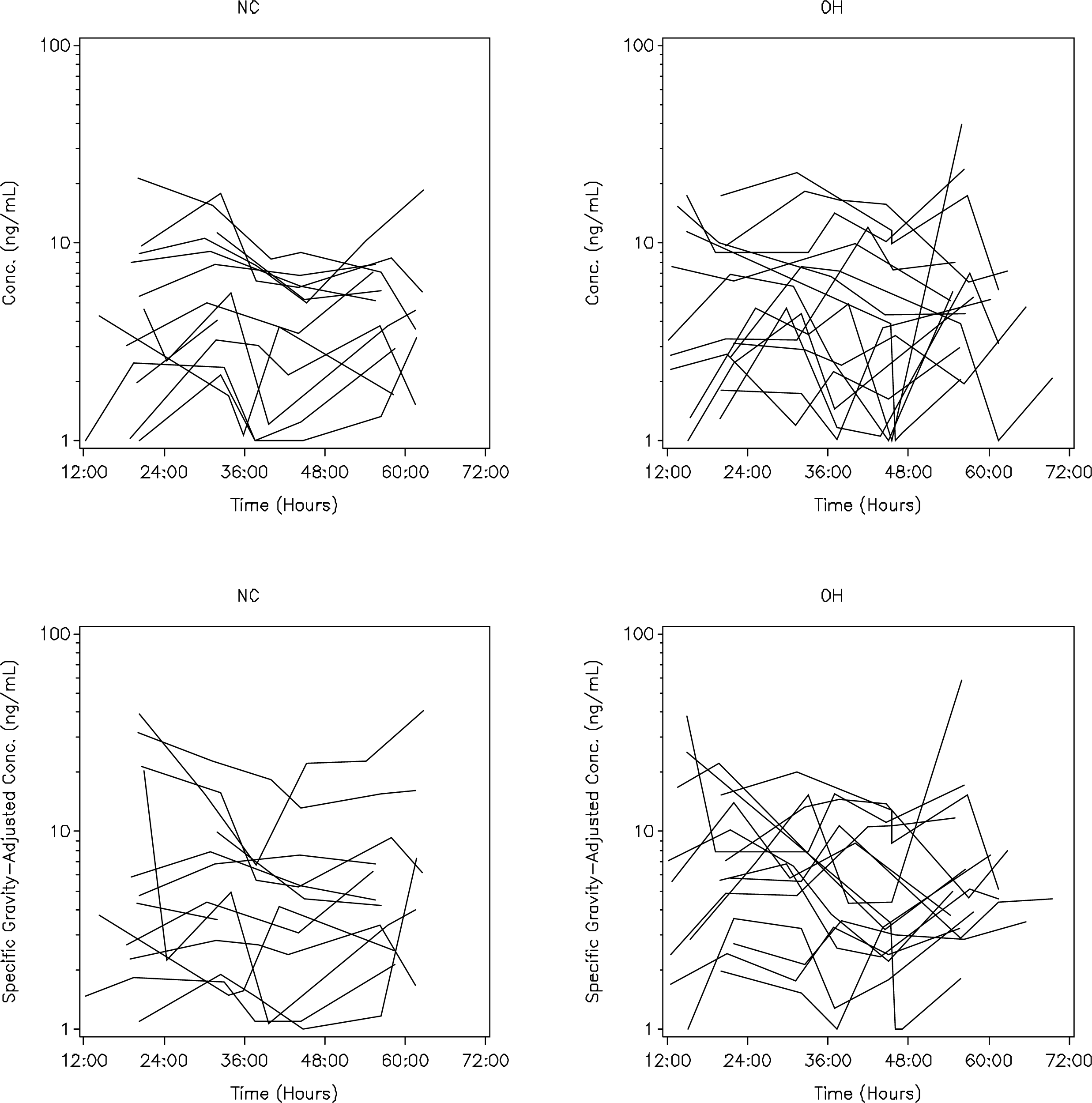

Supplement: Figure S-1. — Concentration versus time plots for urinary TCPy measurements among CTEPP-NC and CTEPP-OH participants reporting a recent pesticide application. Urines in panels A and B are without adjustment. Urines in panels C and D are adjusted by specific gravity. Note that not all voids within the 48 hour period were collected. [file ijerph-08-01727fs1.tif]
